# Diffuse intestinal ulcerations: Diagnostic challenge in a patient with complicated celiac disease

**DOI:** 10.1002/deo2.140

**Published:** 2022-06-20

**Authors:** Mirjana Kalauz, Silvija Cukovic Cavka, Viktor Domislovic, Kiarash Pourmodjib, Matija Kalauz, Snjezana Dotlic, Katja Grubelic Ravic, Zeljko Krznaric

**Affiliations:** ^1^ Department of Internal Medicine Division of Gastroenterology, Zagreb University Hospital Center School of Medicine University of Zagreb Zagreb Croatia; ^2^ School of Medicine University of Zagreb Zagreb Croatia; ^3^ Department of Pathology and Cytology Zagreb University Hospital Center, School of Medicine University of Zagreb Zagreb Croatia

**Keywords:** capsule endoscopy, complicated celiac disease, diffuse intestinal ulcerations, enteropathy‐associated T‐cell lymphoma, enteroscopy

## Abstract

A 48‐year‐old female patient presented with longstanding unrecognized celiac disease (CD), a family history of CD, and a short duration of alarming symptoms. The diagnostic evaluation revealed the concomitant presence of small and large bowel ulcers raised a dilemma about differential diagnosis in her case. Pathologic examination of tissue specimens from the jejunal ulcer led to the diagnosis of enteropathy‐associated T‐cell lymphoma. In recent years, the availability of modern cross‐sectional imaging and endoscopy modalities has dramatically improved the detection and characterization of small bowel lesions. Characterization of small bowel ulcers by endoscopy and radiology imaging in a patient with suspected complicated CD (CCD) needs to be made in conjunction with all clinical factors, as there is a wide overlap of the possible etiologic factors. Enteropathy‐associated T‐cell lymphoma is a highly aggressive T‐cell lymphoma with a poor prognosis, since early diagnosis and appropriate treatment may be delayed due to nonspecific clinical and endoscopic presentation. Therefore, it is crucial to timely recognize patients with suspected CCD and properly navigate diagnostic imaging tools, acquire adequate biopsy, and perform immunophenotyping to set early diagnosis in patients with diffuse intestinal ulcers and CD.

## INTRODUCTION

Celiac disease (CD) is an autoimmune enteropathy caused by gluten exposure in genetically predisposed individuals, characterized by intraepithelial lymphocytosis, crypt hyperplasia, and villous atrophy of small bowel mucosa. There has been a substantial increase in the number of CD diagnoses in recent years, while many patients still remain undiagnosed.^1^ A strict gluten‐free diet (GFD) for life usually leads to small bowel healing and resolution of intestinal and extraintestinal symptoms, making CD a benign disorder with a good prognosis. Delay in diagnosis, age at diagnosis ≥50 years, inadequate response to GFD, and low dietary compliance may increase the risk of complications.^1^

Although rare (around 1% of patients diagnosed with CD), the complications of CD include hyposplenism, refractory CD (RCD), intestinal lymphoma, small bowel adenocarcinoma, and ulcerative jejunoileitis.^1^ The patients are diagnosed with RCD when symptoms persist or recur despite strict GFD for more than 12 months and other causes of villous atrophy have been excluded.^1^ There are two subtypes of RCD depending on the presence or absence of aberrant intraepithelial lymphocytes, which are clonal T‐cells. Type 1 RCD usually develops sooner and has a relatively benign course, whereas type 2 RCD is known to be associated with the highest risk (80%) of progression to enteropathy‐associated T‐cell lymphoma (EATL) and ulcerative jejunitis.^2^, ^3^ Those patients need to be treated in referral centers by a gastroenterologist experienced in CD in collaboration with a hematologist.

Duration and dose of gluten exposure appear to be risk factors for RCD and EATL. Patients with persistent villous atrophy have a significantly higher risk of lymphoproliferative malignancy compared to those with mucosal healing, so it is important to emphasize that in most cases, EATL does not develop from well‐treated CD but rather in the setting of type 2 RCD 2.^4^, ^5^

Herein, we report a case of longstanding, unrecognized CD complicated with EATL in a 48‐year‐old female patient. We also discuss challenges in the diagnostic assessment of patients with suspected CD complications.

## CASE REPORT

**FIGURE 1:**
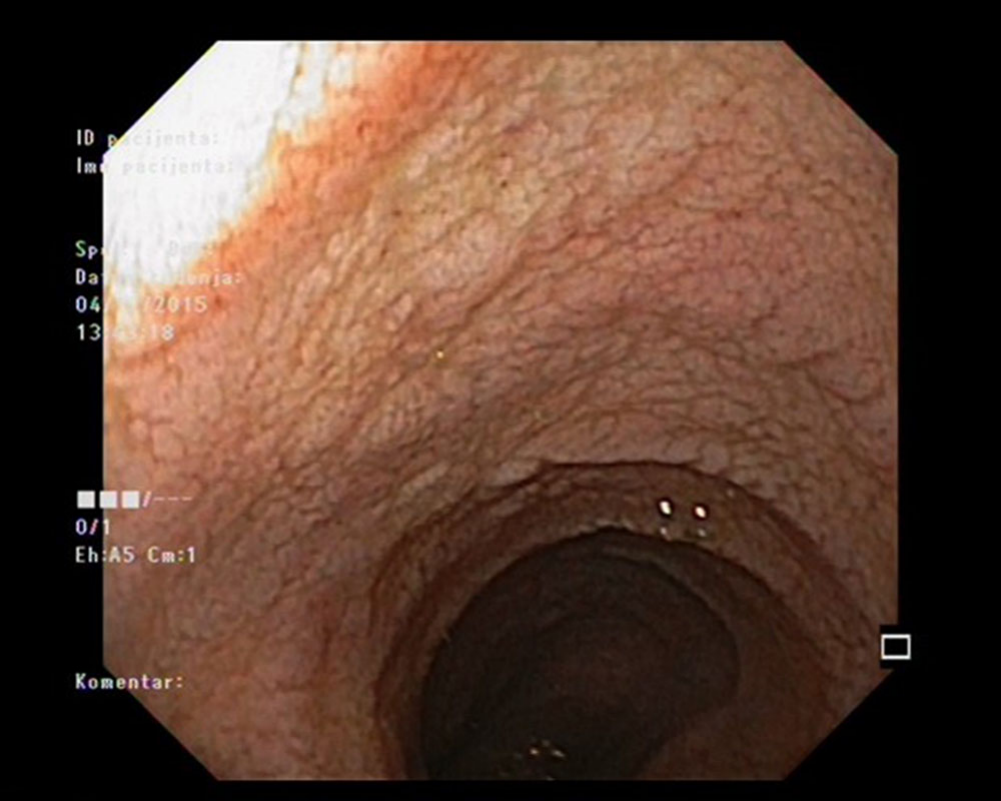
Endoscopic image of duodenal mucosa showing loss of folds with the granular appearance of the mucosa

A 48‐year‐old female patient presented to our gastroenterology outpatient clinic with a 1‐month history of diffuse abdominal pain and significant weight loss. Her family history was strongly positive for CD as her three siblings had CD. Her previous medical history was unremarkable and she had never been tested for CD. Abdominal examination elicited mild epigastric tenderness and her body mass index was at the lower limit of normal. Initial laboratory studies revealed discrete thrombocytosis (497 × 10^9^/L, reference range 158–424 × 10^9^/L), normal values of inflammatory parameters (C‐reactive protein [CRP] 2 mg/L, reference range <5 mg/L), and positive serology for CD (tTg‐IgA >200 RU/ml, reference range <20 RU/ml). Esophagogastroduodenoscopy revealed scalloping of folds and fissuring of intervening duodenal mucosa (Figure 1). Histology showed villous atrophy and lymphocyte infiltration (Marsh 3 lesion); therefore, the diagnosis of CD was confirmed and the patient started GFD.

**FIGURE 2:**
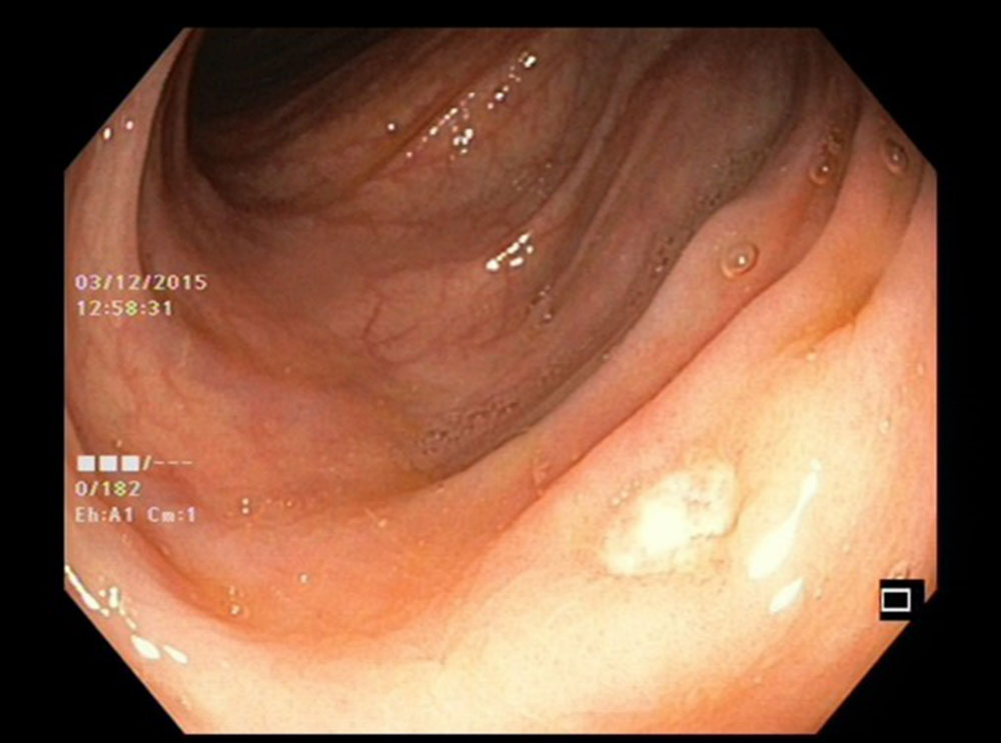
Colonoscopic image showing ulceration in the left colon

**FIGURE 3:**
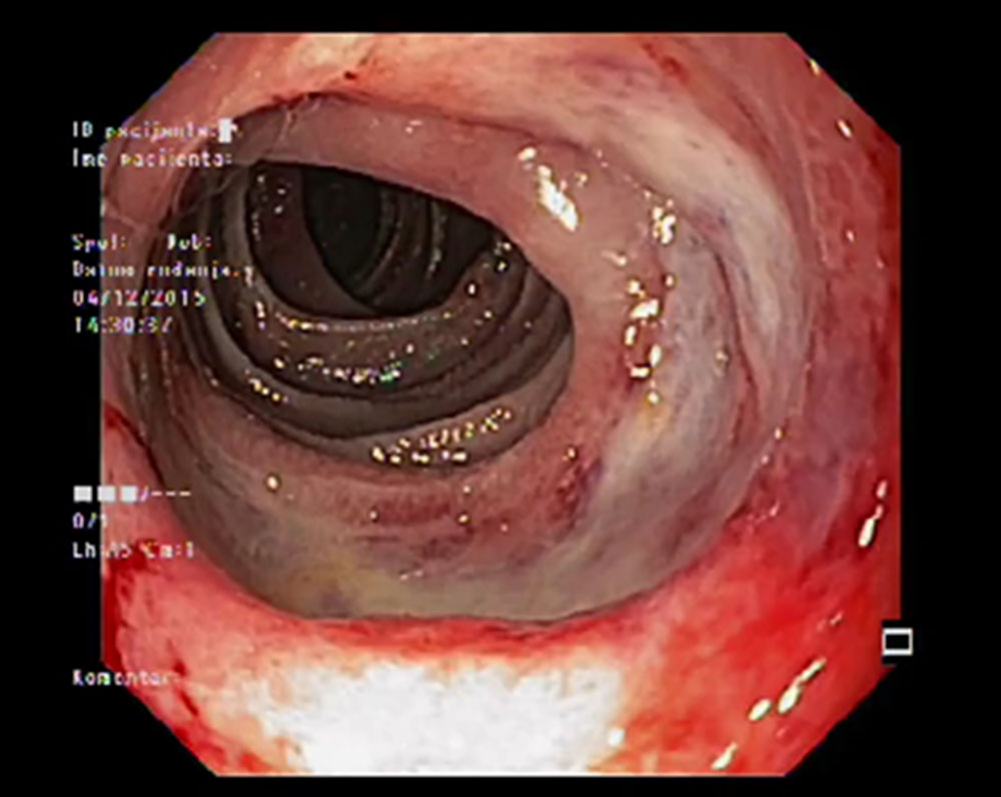
Single‐balloon enteroscopy showing large ulceration in the proximal jejunum

**FIGURE 4:**
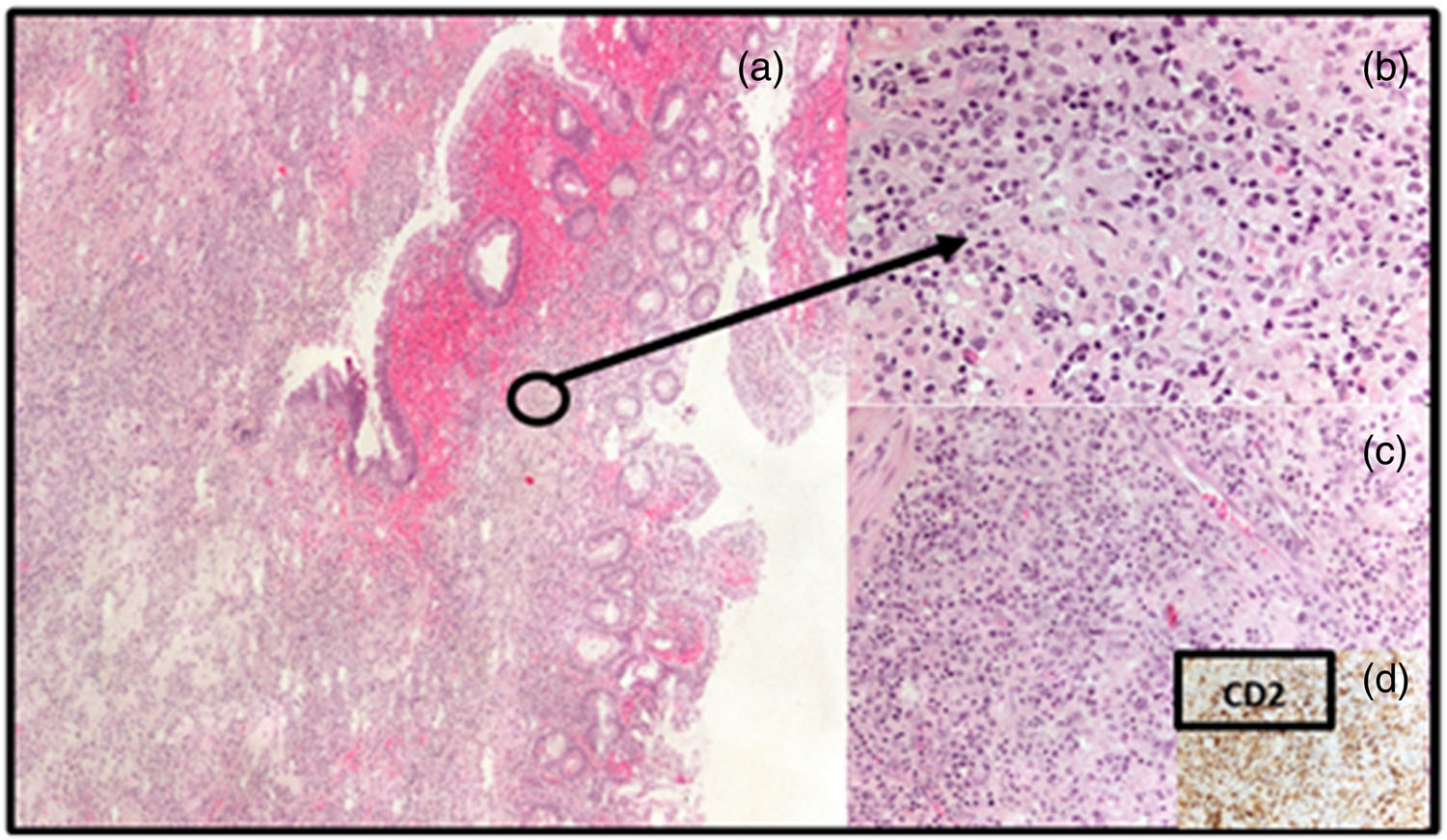
Histopathologic examination of jejunal ulcer biopsy specimen: (a) low‐power microphotograph showing blunting of intestinal villi and ulceration of the mucosal surface; (b) lamina propria infiltrate is composed of atypical medium‐sized and large lymphocytes with focally prominent nucleoli (HE, ×200); (c) neoplastic lymphocytes display significant pleomorphism and abundant cytoplasm (HE, ×400); (d) atypical T‐cells show expression of CD2 (immunohistochemistry CD2, ×200)

During the 2‐month follow‐up, the patient presented with worsening abdominal pain and intermittent fever (38.5˚C), so she was admitted to our gastroenterology department for re‐evaluation. Laboratory workup revealed increased inflammatory parameters (CRP 31.3 mg/L, reference range <5 mg/L) and hypoalbuminemia (serum total albumins 22 g/L, reference range 41–48 g/L), with negative blood cultures. Computerized tomography revealed multiplied and enlarged mesenteric lymph nodes. Lower endoscopy showed multiple ulcerations in all segments of the large bowel with normal surrounding mucosa (Figure 2). Histology report on colon ulcers described mild active inflammation. Small bowel follow‐through (SBFT) revealed a suspicious ulcer in the mid‐small intestine. Therefore, a single balloon enteroscopy was performed to reveal multiple small ulcerations in the distal jejunum and deep, large ulceration in the proximal jejunum with a flat base and thickened margins consuming almost complete circumference of the bowel wall (Figure 3) (Video 1). The endoscopic finding was nonspecific and differential diagnosis included several conditions, i.e. ulcerative jejunoileitis with CD, a lymphoproliferative disorder such as EATL, the concomitant presence of Crohn's disease and CD, and cryptogenic multifocal ulcerous stenosing enteritis. Jejunal ulcer biopsies were obtained and histology indicated medium‐size to large neoplastic T‐immunophenotype lymphocytes with hyperchromatic nuclei and prominent nucleoli (CD3+, CD2+, CD5+/−, CD7+/−, CD8, CD56‐) with enteropathic changes in the background mucosa (Figure 4).

The diagnosis of EATL (stage II2 A) as the first presentation of CD was established. The patient was treated with chemotherapy but during the further clinical course, she developed lethal complications and died 6 months after establishing the diagnosis of EATL.

## DISCUSSION

The prevalence of CD is increasing in Western countries for reasons that are currently unknown.^1^ Some studies have shown that most CD cases remain undiagnosed due to heterogeneous symptoms and poor disease awareness.^1^ Serological testing for CD among individuals with only subtle or atypical symptoms and in risk groups is a favored strategy to increase the detection of CD.^6^ Current guidelines do recommend that serological testing for CD should be performed in different clinical situations, ranging from the presence of potential symptoms of the disease (diarrhea, failure of children to thrive, gastrointestinal symptoms, prolonged fatigue, weight loss, and anemia) through the presence of associated conditions (type 1 diabetes, autoimmune thyroid disease, dermatitis herpetiformis, irritable bowel syndrome).^6^ The prevalence of CD is higher in first‐degree relatives (10%–15%), so active screening in family members of a patient is strongly recommended.^6^

Our patient with longstanding unrecognized silent CD and strong family history presented with a short duration of alarming symptoms that were recognized as signs of complicated disease and prompted further evaluation. During the endoscopic diagnostic evaluation, ileocolonoscopy revealed multiple colon ulcers and histology showed nonspecific findings.

At this point, we considered several small bowel imaging procedures that would be valuable in this setting. According to recent guidelines, capsule endoscopy is considered a first‐line imaging procedure for small bowel investigation.^7^ It has the potential for direct visualization of mucosa throughout the small bowel with noninvasive technology. Furthermore, capsule endoscopy has an excellent diagnostic yield for mucosal lesions in CD patients with suspected complications, with an 8%–14% rate of neoplasia detection.^8^ The most important limitation of capsule endoscopy is the impossibility to take biopsy specimens. Also, there is a considerable risk of capsule endoscopy retention in an obstructive lesion, which could not be excluded in our patient. Enteroscopy is a valuable tool for the management of symptomatic or at‐risk CD patients and gives the possibility of tissue sampling of suspected lesions.^9^, ^10^

As radiologic techniques allow visualization of the entire small bowel, computerized tomography or magnetic resonance enterography may have been the best option for our patient but due to the high cost and lack of expertise, these are not readily available in all centers, including our hospital. Furthermore, positron emission tomography scan has been shown to be effective in the detection of CD‐associated lymphoproliferative disorders such as EATL, and could therefore be suggested in addition to other cross‐sectional imaging techniques in this setting.^7^

So, in order to localize the lesion in the small bowel in our patient, we decided to perform SBFT which revealed an ulcer in the mid‐small intestine. The newer, improved cross‐sectional imaging techniques have mostly replaced fluoroscopic examination as a primary tool for small bowel evaluation. However, some studies suggest that SBFT continues to be used in the setting of inflammatory bowel disease and other disorders.^7^

The results of recent studies suggest that the endoscopic approach has advantages over cross‐sectional imaging investigations in patients with suspected complicated CD (CCD),^9^ but we believe that decision on the imaging modality depends on clinical presentation, probability of obstructive small bowel lesion, need for more accurate assessment of lesion localization, and tissue sampling. Cross‐sectional radiology imaging and endoscopy procedures should be considered complementary methods in the diagnosis and management of patients with suspected CCD.

In our patient, an orthograde single balloon enteroscopy was subsequently performed and revealed jejunal ulcers. Histology was compatible with the diagnosis of EATL, a rare and aggressive complication of CD.

Small and large bowel ulcers in a patient with positive serology and a family history of CD raise a strong suspicion of a complication of untreated CD, although other differentially diagnostic possibilities must be considered.

Three main points of clinical importance are highlighted in this case report. First, early family screening and initiation of GFD are essential for the prevention of neoplastic complications such as EATL. Identification of the subgroup of CD patients at risk deserving an early diagnostic approach is mandatory and even more urgent given the availability of high‐performing imaging tools for small bowel. Second, differentiation of small intestine ulcerations based only on the endoscopic image is not possible as there is a wide overlap, so it is essential to take clinical context into account. Histology is crucial as it can provide diagnostic confirmation. Third, although suitable accuracy for small bowel tumors has been described for radiologic imaging, endoscopy appears to be the most appropriate approach when suspecting early‐stage small bowel tumors.

## CONFLICT OF INTEREST

The authors declare no conflict of interest.

## FUNDING INFORMATION

The corresponding author and the co‐authors are going to overtake the costs.

## HELSINKI DECLARATION

Since the patient died, the Helsinki Declaration is not applicable. The relatives agree with publishing the case report.

## Supporting information


**Video 1**. Single‐balloon enteroscopy showing large ulceration in the proximal jejunum.
